# Synthesis, Characterization and Antifertility Activity of New Unsymmetrical Macrocyclic Complexes of Tin(II)

**DOI:** 10.1155/MBD.2000.237

**Published:** 2000

**Authors:** Kripa Sharma, S. C. Joshi, R. V. Singh

**Affiliations:** 1 Department of Chemistry University of Rajasthan Jaipur 302004 India; 2 Department of Zoology University of Rajasthan Jaipur 302004 India

## Abstract

A new series of unsymmetrical macrocyclic complexes of tin(ll) has been prepared by the template process using bis(3-oxo-2-butylidene)propane-1,3-diamine as precursor. This affords a method to synthesize these complexes with various ring sizes. The tetradentate macrocyclic precursor [N_4_mL] reacts with SnCl_2_ and different diamines in a 1:1:1 molar ratio in refluxing methanol to give complexes of the type [Sn(N_4_mL)Cl_2_]. The ring expansion has been achieved by varying the diamine between the two diacetyl amino nitrogen atoms. The macrocyclic precursor and its metal complexes have been characterized on the basis of elemental analysis, molar conductance, molecular weight determinations, IR, ^1^H NMR,^13^C NMR, ^119^Sn NMR and electronic spectral studies. An octahedral geometry around the metal ion is suggested for these complexes. On the basis of molecular weights and conductivity measurements, their monomeric and non-electrolytic nature has been confirmed. The precursor and complexes have been screened *in vitro* against a number of pathogenic fungi and bacteria to assess their growth inhibiting potential. The testicular sperm density and testicular sperm morphology, sperm motility, density of cauda epididymal spermatozoa and fertility in mating trails and biochemicals parameters of reproductive organs have been examined and discussed.


### SYNTHESIS, CHARACTERIZATION AND ANTIFERTILITY ACTIVITY OF NEW UNSYMMETRICAL MACROCYCLIC COMPLEXES OF TIN(II)

Kripa Sharma[1], S. C. Joshi[2] and R. V. Singh*[1]

[1] Department of Chemistry [2] Department of Zoology, University of Rajasthan, Jaipur 302004, India

### ABSTRACT

A new series of unsymmetrical macrocyclic complexes of tin(II) has been prepared by the template process using bis(3-oxo-2-butylidene)propane-1,3-diamine as precursor. This affords a method to synthesize these complexes with various ring sizes. The tetradentate macrocyclic precursor [$N_4$mL] reacts with $SnCl_2$ and different diamines in a 1:1:1 molar ratio in refluxing methanol to give complexes of the type [Sn($N_4$mL)$Cl_2$]. The ring expansion has been achieved by varying the diamine between the two diacetyl amino nitrogen atoms. The macrocyclic precursor and its metal complexes have been characterized on the basis of elemental analysis, molar conductance, molecular weight determinations, IR, $^1$H NMR,$^{13}$C NMR, $^{119}$Sn NMR and electronic spectral studies. An octahedral geometry around the metal ion is suggested for these complexes. On the basis of molecular weights and conductivity measurements, their monomeric and non-electrolytic nature has been confirmed. The precursor and complexes have been screened *in vitro* against a number of pathogenic fungi and bacteria to assess their growth inhibiting potential. The testicular sperm density and testicular sperm morphology, sperm motility, density of cauda epididymal spermatozoa and fertility in mating trails and biochemicals parameters of reproductive organs have been examined and discussed.

### INTRODUCTION

The coordination chemistry of macrocyclic precursors is a fascinating area which has attracted the attention of inorganic chemists. Macrocyclic polyamines complexes of bivalent transition metals have been of great interest due to their importance as an essential metalloenzyme active site[1] and this will help in furthering our understanding of biological systems[2]. These precursors are also of theoretical interest as they are capable of furnishing an environment of controlled geometry and precursor filed strength[3]. The successful application of several 1, 4, 7, 10-tetraazacyclododecane (cyclen) precursors to the synthesis of macrocyclic complexes stems mainly from their use as models for protein- metal binding sites in biological systems[4] and as selective complexing agents for metal ion[5], such as therapeutic reagents for the treatment of metal toxicity[7,8]. Nowadays interest is focussed on the synthesis of macrocyclic complexes with potential medicinal applications, as contrast-enhancing agents in magnetic resonance imaging (MRI)[9,10] as n.m.r. shift and relaxation reagents[11,12] and as RNA cleavage catalyst[13,14].

Organotin compounds exhibits a broad spectrum of biological activity which includes bactericidal, fungicidal[15] antitumor[16] and caricidal derivatives[17]. Our ongoing work of tin(II) derivatives involving such systems led us to describe the synthetic and stereochemical features of some diorganotin complexes. The biochemistry of synthetic organometallic has generated active research relating to their biochemical significance[18]. The importance of metal-nitrogen bonding and their prominence in agricultural, medicinal and industrial chemistry led us to synthesize and screen the precursors and their macrocyclic compounds for their antifungal, antibacterial and antifertility activities.

### MATERIALS AND METHODS

All reagents were obtained commercially and by standard procedures. All solvents were of reagent grade. $SnCl_2$ was from Sarabhai and Glaxo make. The reactions were carried out under strictly anhydrous conditions.

**Preparation of bis(3-oxo-2-butylidene)propane-1,3-diamine**

In a 100 ml short necked round bottomed flask, diacetyl was taken in ethanol and to this was added 1,3-diaminopropane in ethanol. The reaction was carried out in 2:1 molar ratio heated under reflux for 12 hours. The reaction mixture was cooled and the reddish brown compound obtained was recrystallised from ethanol (Yield 75%).

**Preparation of complexes**

The reaction mixture containing bis(3-oxo-2-butylidene)propane-1,3-diamine, diamine and tin chloride in 1:1:1 ratio in methanol was heated under reflux for 36 hours. The reaction mixture was cooled, transferred to an evaporating dish and set aside for a few hours, whereupon a dark coloured compound separated out. The product formed was washed and dried under reduced pressure, which was recrystallised from a 1:1 mixture of toluene and n-hexane in 78% yield. The physical properties and analytical data are summarized in Table I.

**Table I: Physical Properties and Analytical Data of Precursor and its Compounds**

| Compound | Colour | M.P. (°C) | Analysis(%) Found (Cacd.) M | N | Cl | C | H | Mol.Wt. Found (Calcd) |
|---|---|---|---|---|---|---|---|---|
| $C_{11}H_{18}N_2O_2$ | Reddish Brown | 167-169 | - | 13.11 (13.32) | - | 62.88 (62.83) | 8.61 (8.63) | 186.23 (210.28) |
| $[Sn(C_{17}H_{22}N_4)Cl_2]$ | Brown | 210-213 | 25.33 (25.15) | 11.95 (11.87) | 14.99 (15.02) | 43.35 (43.26) | 4.67 (4.70) | 459.05 (471.98) |
| $[Sn(C_{16}H_{23}N_5)Cl_2]$ | Orange | 198-200 | 25.12 (24.99) | 14.63 (14.74) | 14.79 (14.93) | 40.83 (40.45) | 4.84 (4.88) | 443.92 (474.98) |
| $[Sn(C_{15}H_{27}N_5)Cl_2]$ | Red | 260-262 | 25.60 (25.42) | 14.88 (15.00) | 15.05 (15.18) | 38.56 (38.58) | 5.80 (5.83) | 438.98 (466.99) |
| $[Sn(C_{21}H_{24}N_4)Cl_2]$ | Dark Brown | Above 300 | 22.80 (22.73) | 10.82 (10.73) | 13.43 (13.57) | 48.29 (48.30) | 4.60 (4.63) | 494.59 (522.13) |

**Analytical Methods and Physical Measurements**

Conductivity measurements in dry DMF were performed with a Systronics conductivity bridge type 305 and molecular weights were determined by the Rast Camphor method. IR spectra were recorded on Perkin - Elmer 577 Grating Spectrophotometer. $^1$HNMR spectra were recorded on a Jeol FX-90Q Spectrometer in methanol and DMSO-$d_6$ using TMS as internal standard. Electronic spectra were recorded on a Hitachi U-2000 Spectrophotometer. $^{13}$C NMR spectra were recorded in methanol, using TMS as the standard and $^{119}$Sn NMR spectra were recorded on a Jeol FX-90Q Spectrometer at 33.35 MHz. The chemical shifts were determined relative to the external reference tetramethytin. Nitrogen and chlorine were estimated by Kjeldahl's and Volhard's method, respectively. Tin was estimated gravimetrically as $SnO_2$. Carbon and hydrogen analyses were performed at CDRI, Lucknow.

**BIOLOGICAL STUDIES**

Bactericidal and fungicidal activities data of precursor and its respective complexes against pathogenic bacteria and fungi are given in Tables II and III. A culture of the test organisms was grown on PDA media (Starch, glucose, agar-agar and water) for fungi and agar media (Peptone, beef extract, agar-agar, NaCl and water) for bacteria for several days at the optimum temperature for growth. All the glassware used were sterilized in an autoclave before use. The radial growth method[19] and paper disc-plate method[20] were employed to evaluate the fungicidal (at 25 ± 1°C) and bactericidal (at 30 ± 1°C) activities, respectively.

**Table II: Fungicidal Screening Data of Precursor and its Compounds (percentage inhibition after 96 h)**

| Compound | *Fusarium oxysporum* | | | *Macrophomina phaseolina* | | |
|---|---|---|---|---|---|---|
| Conc. | 50 | 100 | 200 | 50 | 100 | 200 |
| $C_{11}H_{18}N_2O_2$ | 33 | 46 | 56 | 30 | 42 | 47 |
| $[Sn(C_{17}H_{22}N_4)Cl_2]$ | 47 | 65 | 74 | 42 | 53 | 60 |
| $[Sn(C_{16}H_{23}N_5)Cl_2]$ | 66 | 70 | 78 | 52 | 66 | 71 |
| $[Sn(C_{15}H_{27}N_5)Cl_2]$ | 34 | 41 | 69 | 39 | 47 | 58 |
| $[Sn(C_{21}H_{24}N_4)Cl_2]$ | 71 | 77 | 84 | 59 | 69 | 81 |
| Standard (Bavistin) | 86 | 100 | 100 | 82 | 100 | 100 |

**Table III: Antibacterial Activity of Precursor and its Compounds [percentage inhibition (mm) after 24 h] (Conc. in ppm)**

| Compound | *Escherichia coli (-)* | | *Staphylococus aureus (+)* | |
|---|---|---|---|---|
| Conc. | 500 | 1000 | 500 | 1000 |
| $C_{11}H_{18}N_2O_2$ | 3 | 5 | 4 | 6 |
| $[Sn(C_{17}H_{22}N_4)Cl_2]$ | 6 | 7 | 8 | 9 |
| $[Sn(C_{16}H_{23}N_5)Cl_2]$ | 9 | 10 | 11 | 12 |
| $[Sn(C_{15}H_{27}N_5)Cl_2]$ | 4 | 6 | 5 | 7 |
| $[Sn(C_{21}H_{24}N_4)Cl_2]$ | 11 | 12 | 13 | 15 |
| Standard (Streptomycin) | 17 | 18 | 15 | 17 |

**Antifertility Activity**

Male *Wistar* strains rats obtained from ICMR, New Delhi were used. Animals were housed in steel cages and maintained under standard conditions (12 h light/12 h dark cycle; 25 ± 3°C; 35-60% humidity). They were maintained on standard diet (Hindustan Lever Ltd., Mumbai) and water was provided *ad libitum*. Prove fertile male rats were taken and provided into six groups of six each. The group A served as vehicle (olive oil) treated control. Animals of group B and C received starting material and precursor (50 mg/kg b. wt. orally) suspended in olive oil for a period of 60 days. The animals of groups D, E and F received same dose of its [Sn($C_{17}H_{22}N_4$)$Cl_2$], [Sn($C_{17}H_{23}N_5$)$Cl_2$] and [Sn($C_{15}H_{27}N_5$)$Cl_2$] complexes, respectively for the same period. On day 61, testes, epididymis, seminal vesicle and ventral prostate were removed, fat and connective tissue cleared off and kept at –20°C until assayed for total protein, sialic acid, total cholesterol, glycogen and fructose by using standard laboratory techniques.

**Fertility Test**

The mating exposure tests of all animals were performed from day 55 to day 60. They were cohabited with proestrous females in ratio 1:3. The vaginal plug and the presence of sperm in the vaginal smear were checked for positive mating. The mated females were separated to note the implantation sites on day 16 of pregnancy through leprotomy.

### RESULTS AND DISCUSSION

The elemental analyses and analytical data of the prepared complexes are given in Table I. All the complexes are stable at room temperature and non-hygroscopic. The products so obtained are soluble in common organic solvents, DMF and DMSO. Complexes have been found to be monomeric as evidenced by their molecular weight determinations. The low values of their molar conductivities (14-26 $\Omega^{-1}$ $cm^2$ $mol^{-1}$) in anhydrous dimethylformamide show them to be non-electrolytes.

***Spectral Studies***

**IR Spectra**

The infrared spectra of the precursor and its tin complexes were recorded and important features may be summarized as follows. In the IR spectra of the complexes, the stretching and deformation vibrations of any $NH_2$ signal are absent, indicating the formation of complexes[21]. Strong bands appearing in the range 1615-1600 $cm^{-1}$ are assigned[22] to the coordinated υC=N stretching vibrations in all the complexes. Two distinct bands of the methyl moiety occurring at 2962 $cm^{-1}$ ($\upsilon_{as}$ $CH_3$) and 2865 $cm^{-1}$ ($\upsilon_s$ $CH_3$) are present in all the complexes. Strong and sharp bands in the spectra of the metal complexes for C-H stretching and bending vibrations appear at *ca* 2820 and 1430 $cm^{-1}$, respectively[21].

**$^{1}$H NMR Spectra**

The $^{1}$HNMR spectrum of the precursor does not show any $NH_2$ signal any more indicating that the proposed macrocyclic skeleton has been formed. A singlet observed at δ 3.14 - 3.56 ppm in the complexes may be assigned to methylene protons adjacent to nitrogen. A complex pattern in the region 2.15 – 2.25 ppm was assigned as middle methylene protons of 1,3-diaminopropane moiety. The shift of the signals towards lower field is an identification of the coordination of the precursor. A singlet observed at δ1.23-1.68 ppm in complexes and precursor is attributable to methyl protons. The complex patterns of aromatic protons were observed at δ7.30-8.30 ppm in the spectra of the precursor as well as complexes. Chemical shift values are given in Table IV.

**Table IV: $^{1}$H NMR Data (δ, ppm) of Precursor and its Compounds (in DMSO-$d_6$)**

| Compound | >N-$CH_2$(bs) | R | | -$CH_3$(s) | >NH | -$CH_2$- |
|---|---|---|---|---|---|---|
| $C_{11}H_{18}N_2O_2$ | 3.14 (4H) | - | | 1.23 (12H) | - | 2.13 (2H) |
| [Sn($C_{17}H_{22}N_4$)$Cl_2$] | 3.45 (4H) | 8.15($H_{2,5}$ d) | 7.30($H_{3,4}$ d) | 1.57 (12H) | - | 2.19 (2H) |
| [Sn($C_{16}H_{23}N_5$)$Cl_2$] | 3.36 (4H) | 8.00($H_{2,4}$ s) | 7.38 ($H_3$ d) | 1.68 (12H) | - | 2.05 (2H) |
| [Sn($C_{15}H_{27}N_5$)$Cl_2$] | 3.40 (12H) | - | - | 1.28 (12H) | 4.33 | 2.16 (2H) |
| [Sn($C_{21}H_{24}N_4$)$Cl_2$] | 3.56 (4H) | 8.3($H_{2,7}$ b) | 7.30 ($H_{3,6}$ d)<br>7.28 ($H_{4,5}$ d) | 1.46 (12H) | - | 2.10 (2H) |

**$^{1}$HNMR Data (δ, ppm) of Precursor and its Compounds (in methanol)**

| Compound | >N-$CH_2$(bs) | R | | -$CH_3$(s) | >NH | -$CH_2$- |
|---|---|---|---|---|---|---|
| $C_{11}H_{18}N_2O_2$ | 2.61 (4H) | - | | 1.02 (12H) | - | 1.91 (2H) |
| [Sn($C_{17}H_{22}N_4$)$Cl_2$] | 2.94 (4H) | 7.63 ($H_{3,6}$d) | 6.78 ($H_{4,5}$d) | 1.05 (12H) | - | 1.83 (2H) |
| [Sn($C_{16}H_{23}N_5$)$Cl_2$] | 2.79 (4H) | 7.42 ($H_{4,6}$s) | 6.88 ($H_5$d) | 1.10 (12H) | - | 2.09 (2H) |
| [Sn($C_{15}H_{27}N_5$)$Cl_2$] | 2.84 (12H) | - | 6.74 ($H_{3,6}$d) | 1.01 (12H) | 3.92 | 2.11 (2H) |
| [Sn($C_{21}H_{24}N_4$)$Cl_2$] | 3.05 (4H) | 7.76 ($H_{2,7}$b) | 6.72 ($H_{3,6}$b)<br>6.70 ($H_{4,5}$d) | 1.20 (12H) | - | 1.98 (2H) |

**$^{119}$Sn NMR Spectra**

The $^{119}$Sn NMR spectrum of the complex [Sn($C_{15}H_{27}N_5$)$Cl_2$] shows the signal at δ-568.96 ppm which gives good agreement with a hexacoordinated tin[23].

**$^{13}$C NMR Spectra**

The $^{13}$C NMR spectra of the unsymmetrical complexes are comparable to those of their precursor and the assigned peaks position are listed in Table V. The shift of carbons attached to nitrogen indicate the involvement of these atoms in coordination and support the formation of macrocyclic framework, as reported earlier also[24,25].

**Table V: $^{13}$C NMR Spectral Data (δ, ppm) of Precursor and its Compounds**

| Compound | >C=O | >N-CH$_2$ | >C=N | -CH$_3$ | R | -CH$_2$- |
|---|---|---|---|---|---|---|
| $C_{11}H_{18}N_2O_2$ | 168.34 | 40.89 | 151.98 | 12.30 | - | 28.52 |
| $[Sn(C_{17}H_{22}N_4)Cl_2]$ | - | 43.54 | 162.04 | 16.82 | $C_{1,6}$ 129.74; $C_{2,5}$ 127.21; $C_{3,4}$ 125.82 | 31.78 |
| $[Sn(C_{16}H_{23}N_5)Cl_2]$ | - | 45.28 | 158.45 | 11.26 | $C_{1,5}$ 136.60; $C_{2,4}$ 125.09; $C_3$ 124.38 | 26.01 |
| $[Sn(C_{15}H_{27}N_5)Cl_2]$ | - | 40.96 | 165.73 | 19.02 | - | 33.05 |
| $[Sn(C_{21}H_{24}N_4)Cl_2]$ | - | 44.48 | 160.82 | 12.40 | $C_{2,7}$ 128.63; $C_{3,6}$ 127.83; $C_{4,5}$ 126.71 | 35.92 |

**Electronic Spectra**

The electronic spectra of the complexes were recorded in distilled DMSO. The absorption maximum appear at 423 nm in the case of precursor can be assigned to the n-π* transitions of the azomethine group. This band is shifted by 10-15 nm, which is consistent with the nitrogen coordination of macrocyclic precursor[26]. In addition to this band, the spectra of the metal complexes exhibit bands around 295 nm and 339 nm due to π-π* electronic transitions. However, the position of these remains almost same as that of the precursor.

On the basis of above evidences, structure (*1*) may be proposed.

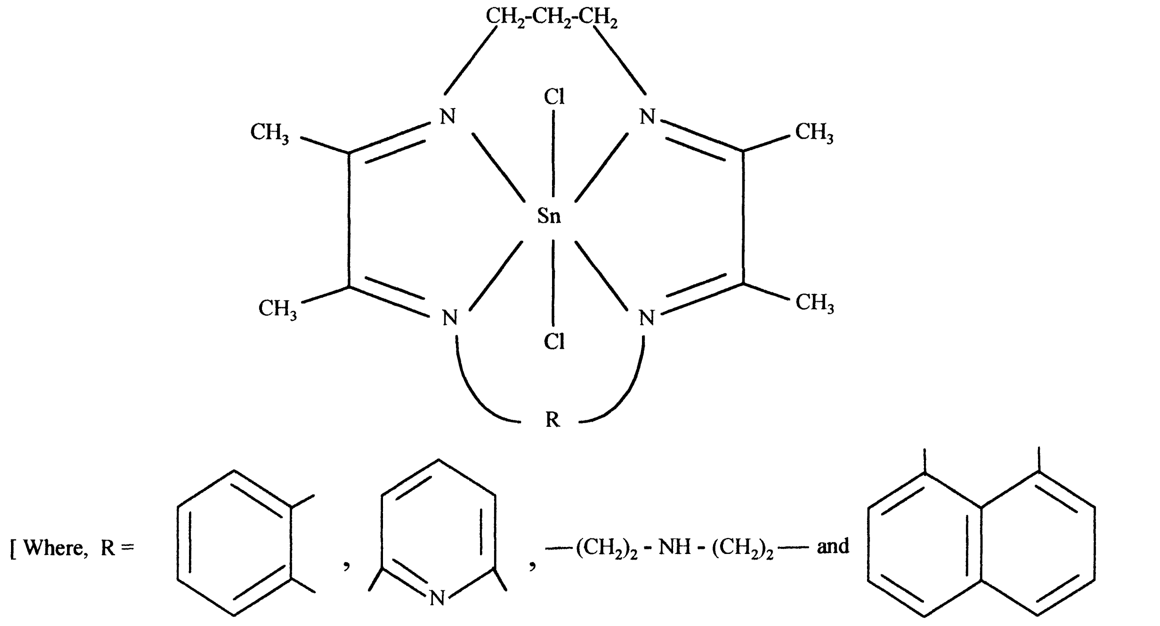

***(1)***

**Biochemical Aspects**

From Tables II and III, it is clear that the toxicity increases as the concentration and complexation increased. The mechanism of the toxicity of these complexes to microorganisms may be due to the inhibition of energy or ATP production[21], for instance by inhibition of respiration or by uncoupling of oxidative phosphorylation. The energy producing processes are located partly in the cytoplasm and partly in the mitochondria. It is apparent from the bactericidal activity towards *Staphylococcus aureus* as compared to *Escherichia coli*. The reason is the difference in the structure of the cell walls of organisms with (+) and (-) strains, respectively. The walls of Gram(-) cells are more complex than those of Gram (+) cells. The lipopolysacchride forms an outer lipid membrane and contributes to the complex antigenic specificity of Gram(-) cells.

Starting material, precursors and their complexes treatment did not alter the body weights of rats. However, the weights of testes, epididymis, seminal vesicle and ventral prostate were reduced significantly. (Table VI )

**Table VI : Changes in the Body Weight of Reproductive Organs after Treatment with Various Compounds**

| Group | Treatment | Body Weight (g) | | Testes | Epididymis | Ventral Prostate | Seminal Vesicle |
|---|---|---|---|---|---|---|---|
| | | Initial | Final | Mg/100 gm b. wt. | | | |
| A | Control | 240±15 | 260±10$^c$ | 1350±45 | 600±10 | 320±10 | 650±15 |
| B | $C_4H_6O_2$ | 235±8 | 245±10$^c$ | 1120±20$^a$ | 510±20$^a$ | 280±15$^c$ | 550±10$^a$ |
| C | $C_{11}H_{18}N_2O_2$ | 220±9 | 230±12$^c$ | 1050±30$^b$ | 450±10$^b$ | 200±15$^b$ | 525±12$^b$ |
| D | $[Sn(C_{17}H_{22}N_4)Cl_2]$ | 245±11 | 250±14$^c$ | 1020±20$^b$ | 430±15$^b$ | 190±10$^b$ | 503±8$^b$ |
| E | $[Sn(C_{16}H_{23}N_5)Cl_2]$ | 238±10 | 246±8$^c$ | 905±30$^b$ | 410±10$^b$ | 170±5$^b$ | 500±15$^b$ |
| F | $[Sn(C_{15}H_{27}N_5)Cl_2]$ | 210±15 | 225±13$^c$ | 900±20$^b$ | 405±15$^b$ | 155±10$^b$ | 450±9$^b$ |

a = $p \leq 0.05$, b = $p \leq 0.001$, c = p = NS. Group B compared with A, Groups C,D,E and F compared with B

**Sperm Concentration /Motility/ Fertility**

Oral administration of various complexes to rats significantly reduced sperm concentration of testes and epididymis. The motility of cauda epididymal sperms was also reduced significantly. Various compounds reduced the fertility of male rats (Table VII).

**Table VII: Sperm Motility, Concentration and Fertility after Treatment with Precursor and Compounds**

| Group | Treatment | Sperm Motility (%) Cauda Epididymis | Sperm Density (Million/ml) | | Fertility (%) |
|---|---|---|---|---|---|
| | | | Testes | Cauda epididymis | |
| A | Control | 74.5±2.4 | 4.30±0.25 | 55.2±3.0 | 100%(+ve) |
| B | $C_4H_6O_2$ | 60.5±7.0$^a$ | 2.9±0.3$^b$ | 45±2.0$^b$ | 85%(-ve) |
| C | $C_{11}H_{18}N_2O_2$ | 45±5.0$^b$ | 2.0±0.02$^b$ | 30±1.0$^b$ | 90%(-ve) |
| D | $[Sn(C_{17}H_{22}N_4)Cl_2]$ | 40±5.3$^b$ | 1.9±0.01$^b$ | 25±2.0$^b$ | 92%(-ve) |
| E | $[Sn(C_{16}H_{23}N_5)Cl_2]$ | 38±3.5$^b$ | 1.1±0.2$^b$ | 20±3.0$^b$ | 95%(-ve) |
| F | $[Sn(C_{15}H_{27}N_5)Cl_2]$ | 35.0±3.2$^b$ | 0.8±0.3$^b$ | 15±2.0$^b$ | 96%(-ve) |

Cf Table VI

**BIOCHEMICAL PARAMETERS**

Protein contents of testes, epididymis, auxiliary glands (seminal vesicle and ventral prostate) were reduced significantly after treatments with various compounds as compared to controls. Sialic acid contents of the testes, epididymis, seminal vesicle and ventral prostate were depleted (Table VIII). Testicular glycogen, cholesterol and seminal vesicular fructose were decreased significantly (Table IX).

**Table VIII: Effects of Various Compounds on Total Protein and Sialic acid Contents of Various Reproductive Organs of Male Rats**

| Group | Treatment | Total Protien (mg/gm) | | | | Sialic Acid (mg/gm) | | | |
|---|---|---|---|---|---|---|---|---|---|
| | | Testes | Epididymis | Ventral Prostate | Seminal Vesicle | Testes | Epididymis | Ventral Prostate | Seminal Vesicle |
| A | Control | 180±5 | 275±10 | 170±8 | 190±10 | 4.5±0.3 | 6.0±0.1 | 5.3±0.2 | 4.3±0.1 |
| B | $C_4H_6O_2$ | 159±2$^a$ | 215±15$^a$ | 140±5$^a$ | 150±5$^a$ | 3.7±0.1$^a$ | 5.0±0.2$^a$ | 4.5±0.2$^a$ | 3.9±0.2$^a$ |
| C | $C_{11}H_{18}N_2O_2$ | 125±10$^b$ | 180±5$^a$ | 112±3$^a$ | 125±2$^b$ | 2.9±0.2$^b$ | 4.0±0.02$^b$ | 4.3±0.1$^b$ | 3.1±0.1$^b$ |
| D | $[Sn(C_{17}H_{22}N_4)Cl_2]$ | 120±7$^b$ | 150±8$^b$ | 100±3$^b$ | 115±4$^b$ | 2.5±0.1$^b$ | 3.8±0.01$^b$ | 4.2±0.2$^b$ | 3.0±0.2$^b$ |
| E | $[Sn(C_{16}H_{23}N_5)Cl_2]$ | 105±10$^b$ | 130±10$^b$ | 98.0±7$^b$ | 108±7$^b$ | 2.4±0.2$^b$ | 3.9±0.02$^b$ | 4.0±0.1$^b$ | 2.9±0.3$^b$ |
| F | $[Sn(C_{15}H_{27}N_5)Cl_2]$ | 100±15$^b$ | 110±7$^b$ | 100±5$^b$ | 105±8$^b$ | 2.5±0.08$^b$ | 3.5±0.01$^b$ | 3.5±0.2$^b$ | 2.8±0.2$^b$ |

Cf Table VI

The present study indicates that oral administration of various compounds brought about a significant reduction in weight of testes, epididymis, ventral prostate and seminal vesicles. The reduced testicular and sex accessories weight reflect wide spread cellular alteration and androgen depletion in the sex organs. Significant reduction in the sperm motility of cauda epididymis was observed in all experimental groups. This may be due to an interference with enzyme reactions including the oxidation phosphorylation uncoupling[28,29]. Our biochemical studies revealed reduction in testicular glycogen probably due to a decrease of the number of post meiotic germ cells, which are sites of the glucose metabolism[29,30]. Decreased protein contents of testes and sex accessories after administration of these complexes could be correlated with loss of total membrane protein and fall in circulating androgen. Reduced sialic acid contents of seminal vesicle caused degenerative changes on the structural integrity sperm cells[31,32]. Addition of the tin moiety and diamines enhance the activity.

**Table IX: Changes in Tissue Cholesterol, Glycogen and Fructose Contents after Treatment with Various Compounds in Male Rats**

| Group | Treatment | Testicular Cholesterol (mg/gm) | Testicular Glycogen (mg/gm) | Seminal Vesicular Frustose (mg/gm) |
|---|---|---|---|---|
| A | Control | 7.9±0.5 | 4.83±0.5 | 480±18 |
| B | $C_4H_6O_2$ | 6.9±0.03 [b] | 4.0±0.3 [a] | 390±10 [b] |
| C | $C_{11}H_{18}N_2O_2$ | 6.2±0.07 [b] | 3.9±0.05 [b] | 300±15 [b] |
| D | $[Sn(C_{17}H_{22}N_4)Cl_2]$ | 6.0±0.03 [b] | 3.8±0.03 [b] | 250±20 [b] |
| E | $[Sn(C_{16}H_{23}N_5)Cl_2]$ | 5.0±0.07 [b] | 3.0±0.05 [b] | 230±15 [b] |
| F | $[Sn(C_{15}H_{27}N_5)Cl_2]$ | 4.4±0.09 [b] | 3.1±0.03 [b] | 150±10 [b] |

Cf Table VI

Nevertheless, the present results amply demonstrate the effects of these compounds on the male reproductive system of rats and the important endocrine function of biosynthesis and secretion of androgen[35]. Further, the current study strongly demonstrate that compound E and F are more effective in inhibiting fertility.

### ACKNOWLEDGEMENT

The authors are thankful to CSIR, New Delhi, India for financial assistance through grant number 01 (1490)/EMR - II .

### REFERENCES

1. I. Bertini and C. Luchinat, in "Bioinorganic Chemistry", ed. I. Bertini, H.B. Gray, S.J. Lippard and J. Valentine, University *Science Books,* Mill Valley, CA, USA (1994).
2. K.J. Lee and J. Suh, *Bioorg. Chem.,* **22** (1994) 95.
3. R.D. Hancock, G. Pattrick, P.W. Wade and G.D. Hosken, *Pure Appl. Chem.*, **65** (1993) 473.
4. E. Kimura, *Pure Appl. Chem.*, **65** (1993) 355.
5. H. Tsubuke, T. Yoden, T. Iwachido and M. Zenki, *J. Chem. Soc., Chem. Commun.* 1069 (1991).
6. S. Blain,P. Appriou, H. Chaumeil and H. Handel, *Anal. Chem. Acta,* **232** (1990) 331.
7. R.A. Bullman, *Struct. Bonding* (Berlin), **67** (1987) 41.
8. D. Bryce-Smith, *Chem. Soc. Rev.*, **15** (1986) 93.
9. J.L. Bousquet, S. Saini, D.D. Stark, P.F. Hahn, M. Nigam, J. Wittenberg and J. Ferrucci, *J. Radiology,* **166** (1988) 693.
10. M.F. Tweedle, G.T. Gaughan and J.H. Hagan, U.S. Patent 4885363 (1987).
11. D.C. Buster, M.M.C.A. Castro, C.F.G.C. Geraldes, C.R. Mallor, A.D. Sherry and T.C. Siemers, *Magn. Reson. Med.*, **15** (1990) 25.
12. J. Platzek, P. Blaszkiewicz, H. Gries, P.Lugar, G. Michl, A. Miiller Fahrnow, B. Radiichel and D. Siilzle, *Inorg. Chem.,* **36** (1997) 686.
13. S.Aimem, J.R. Morrow,C.R. Lake amd M.R. Churchill, *Angew. Chem.Int. Ed. Engl.,* **33** (1994) 773.
14. B.F. Baker, H.Khalili, N. Wei and J.R. Morrow, *J. Am. Chem.Soc.,***119** (1998) 38.
15. M. Gielen, E.R.T. Tiekink, A. Bouhdid, D. Devos, M. Biesemans, I. Verbruggen and R.William, *Appl. Organomet. Chem.*, **9** (1995) 639.
16. R. William, H. Dalel, P. Brockaert, M. Biesemans, L. Ghys, K. Nooter, D. Vos, F. Ribot and M. Gielen, *Main Group Met. Chem.*, **20** (1997) 535.
17. M. Gielen, R. Willem, J. Holecek and A. Lycka, *Main Group Met. Chem.*, **16** (1993) 29.
18. N. Fahmi, S.C.S. Jadon and R.V. Singh, *Phosphorus, Sulfur and Silicon*, **81** (1993) 140.
19. A. Kumari, J.P. Tondon and R.V. Singh, *Appl., Organomet. Chem.*, 7 (1993) 655.
20. P. Dixit and J.P. Tandon, *Phosphorous, Sulfur and Silicon*, **53** (1990) 389.
21. M. Shakir, S.P. Varkey and T.A. Khan, *Indian J. Chem*, **34A** (1995) 72.
22. M. Shakir, S.P. Varkey and P.S. Hameed, *Polyhedron*, **13** (1994) 1355.
23. M. Veith, S. Mathur and V. Huch, *J. Chem. Soc., Dalton Trans*, (1996) 2485.
24. K. Fujita, M. Ikeda, Y. Nakano, T. Kondo and T. Mitsudo, *J. Chem. Soc., Dalton Trans.*, 1998 (2908).
25. R.S. Silva, M.T.P. Gambardella, R.H.A. Santos, B.E. Mann and E. Tfouni, *Inorg. Chim. Acta*, **245** (1996) 218.
26. K. Singh, P. Dixit, R.V. Singh and J.P. Tanson, *Main Group Met. Chem.*, **12** (1989) 155.
27. J. Horsfall, *Bot. Rev.*, (1945) 419.
28. T. Pandey, V.P. Singh and R.V. Singh, *Main Group Met. Chem.*, **21** (1998) 185.
29. R.W. Marsh, *Systemic Fungicides*, Long man, London, 1972, p. 150.
30. H. Levinsky, R. Singh, M. Garnet, M. Sagiv and D. Allalauf, Sialic acid contents of human spermatozoa and seminal plasma in relation of sperm counts Arch. Androl, **10** (1983) 45.
31. A. Purohit, V.B. Joshi and V.P. Dixit, Contraceptive efficacy of *Azadirachta indica* (Flower and Bark) in male rats: A biochemical and sperm dynamics analysis, *J. Curr. Biosci,* 7 (1990) (4) 129-133.

32. Y. B. Ke and W.W. Tso (1982): Variation of Gossypol succeptibility in rats spermatozoa during spermatogenesis, **27** (1982) (1), 142-146.
33. K.P. Gunaga, M.C. Rao, A.R. Sheth and S.J. Rao, The role of glycogen during the development of rat testes and prostate. *J. Reprod. Fertile,* **29** (1972) 157-132.

**Received: October 16, 2000 - Accepted: October 20, 2000 -**
**Received in revised camera-ready format: November 29, 2000**

